# Preservation Fluid Bacteriology in Kidney Transplantation: Comparing Uncontrolled Donation After Circulatory Death With Donation After Brain Death

**DOI:** 10.3389/ti.2025.14855

**Published:** 2025-07-25

**Authors:** Alberto Costa Silva, Teresa Pina-Vaz, Ana Pinho, Inês Ferreira, Ana Cerqueira, Manuela Bustorff, Susana Sampaio, Roberto Roncon-Albuquerque, Margarida Rios, Manuel Pestana, Carlos Martins-Silva, Tiago Antunes-Lopes, João Alturas Silva

**Affiliations:** ^1^ Urology Department, University Hospital Center of São João, Porto, Portugal; ^2^ Faculty of Medicine, University of Porto, Porto, Portugal; ^3^ RISE- Health Research Network, Faculty of Medicine, University of Porto, Porto, Portugal; ^4^ Nephrology Department, University Hospital Center of São João, Porto, Portugal; ^5^ Intensive Care Unit, University Hospital Center of São João, Porto, Portugal; ^6^ Transplant Coordination Office, University Hospital Center of São João, Porto, Portugal

**Keywords:** kidney transplantation, organ procurement, preservation fluid, donation after circulatory death, infection

## Abstract

Infectious complications remain a significant concern in organ transplantation, and preservation fluid (PF) has been identified as a potential source of microbial contamination. However, the clinical relevance of positive PF cultures, especially in kidney transplants from uncontrolled donation after circulatory death (uDCD), is not clearly established. This study aims to evaluate and compare the incidence and clinical implications of positive PF cultures in kidney transplants from uDCD and donation after brain death (DBD) donors. A prospective, single-center study was conducted, involving 497 kidney transplants—147 from uDCD and 350 from DBD donors. PF samples were systematically collected at the time of transplantation, cultured, and analyzed. The type of bacteria identified guided antibiotic treatment decisions. Recipients were monitored for the development of bacteremia within the first post-transplant week. Positive PF cultures were significantly more frequent in uDCD transplants (32.0%) compared to DBD (13.7%) (p < 0.001). Coagulase-negative staphylococci predominated in both groups. Despite this, bacteremia rates were comparable—8.5% in uDCD and 6.3% in DBD (p = 0.673)—with no culture-concordant cases. Antibiotics were administered to 10.6% of uDCD and 22.9% of DBD recipients (p = 0.110). Although uDCD kidneys had higher PF contamination, the clinical impact was minimal.

## Graphical Abstract

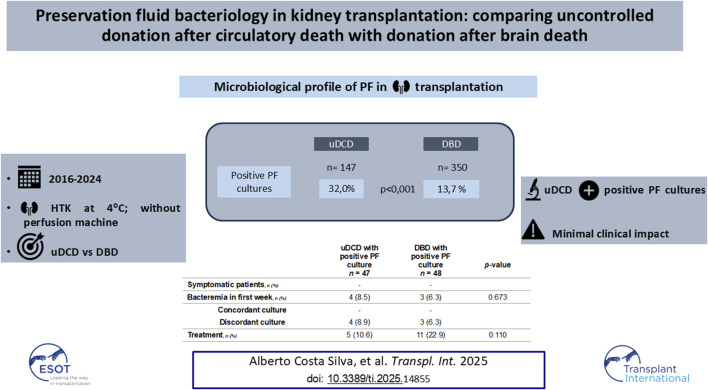

## Introduction

Infectious complications are a significant cause of morbidity and mortality in organ transplant patients [1]. Preservation fluid (PF) is a critical component in maintaining organ viability after procurement but also poses a risk of infection, which can arise from microorganisms originating from the donor or introduced during recovery and handling [2, 3]. Routine screening of PF for microbial growth and the clinical implications of culture-positive results remains contentious issues. The effects on recipients can range from asymptomatic colonization to mild or severe infections, potentially leading to graft failure and even death [4]. The incidence of culture-positive PF in solid organ transplantation has been reported to 7.2%–77.8%, with kidney transplantation alone showing positive cultures in up to 24% of cases, and pathogenic microorganisms identified in 10% [2, 5, 6].

Given the increasing demand for suitable grafts, kidneys from uncontrolled donation after circulatory death (uDCD) with *in situ* preservation using extracorporeal membrane oxygenation (ECMO) have emerged as a viable alternative, thereby expanding the organ donor pool [7, 8]. However, there is currently no data on the microbiological profile of PF in uDCD transplantation, particularly in the context of kidney transplantation.

This study aimed to assess the incidence of positive microbiological cultures in PF from uDCD kidney transplants compared to those from donation after brain death (DBD) and its clinical impact.

## Materials and Methods

A prospective study was conducted at a single center in accordance with the Declaration of Helsinki, following approval by the institutional review board and after obtaining informed consent from all participants. Transplants performed between January 2016 and December 2024 were included in the study. Exclusion criteria comprised transplants from living donors, cases from donors on antibiotics/with suspected infection and cases requiring surgical reintervention within the first 8 days post-transplant.

Transplants were categorized into two groups: uDCD and DBD, with the DBD group including both standard criteria donors (SCD) and expanded criteria donors (ECD).

All donors were from the same center. Donor surgical prophylaxis in the procurement was not made and recipients received single shot of cephazolin or ciprofloxacin in case of allergy. Preoperative skin cleansing was made with povidone-iodine antiseptic solution.

Following organ collection, they were placed in a sterile container, immersed in Custodiol^®^ solution, and maintained at approximately 4°C without perfusion machine. At the time of transplantation, immediately before back-table surgery, a 20 mL sample of PF was collected and sent for culture within 4 h. This liquid was centrifuged and the pellet inoculated in solid media such as: non-selective, blood agar and selective, MacConkey agar and mannitol-salt agar and liquid, Brain-heart broth for 24-48 h at 37°C in aerobic conditions. The liquid media was then inoculated in blood agar and incubated for more 24 h in aerobic conditions. Identification of the colonies and antimicrobial susceptibility assay took place.

Patients were grouped according to whether their PF culture was positive or negative after 72 h of culture. At that time, for culture-positive patients, the type of bacteria was recorded, and an assessment of the recipient’s blood culture was performed. Patients were classified as culture-concordant if the same bacteria was identified in PF and blood cultures, and culture-discordant if not. Patients were classified as symptomatic based on the presence of fever and elevated inflammatory markers, such as leukocyte count or C-reactive protein levels; wound site infection was also considered.

Treatment decisions were based on the bacteria identified in the PF culture; specifically, patients were treated for *Escherichia coli, Enterococcus faecium, Enterococcus faecalis, Staphylococcus aureus, Klebsiella pneumoniae, Klebsiella aerogenes, Acinetobacter baumannii, Pseudomonas aeruginosa*, and *Enterobacter* species. Antibiotic therapy was initiated upon availability of the PF culture results, typically 48–72 h after transplantation.

Demographic data were collected, as well as surgical extraction time (interval between the donor’s surgical incision and kidney’s transfer to cold storage), cold ischemia time (time between aortic clamping and transplantation), cause of chronic kidney disease (CKD) and type of dialysis. Induction immunosuppression for all uDCD and DBD recipients with high immunological risk was carried out using rabbit anti-thymocyte globulin. For DBD recipients without high immunological risk, basiliximab was administered. Maintenance therapy consisted of prednisolone, mycophenolate mofetil, and tacrolimus.

Data collection and analysis were performed using Statistical Package for the Social Sciences version 27 (IBM, Chicago, United States). The Kolmogorov–Smirnov test was used to evaluate the distribution of the parameters. Continuous variables with a normal distribution are presented as the mean ± standard deviation, while non-normally distributed variables are represented using the median and percentiles 25 and 75. The chi-square test was used to analyze categorical variables and to compare categorical and continuous variables, we employed the t-test or Kruskal–Wallis test, depending on the normality of the data. Statistical significance was set at *p* < 0.05.

## Results

PF cultures were analyzed from a total of 497 kidney transplants, including 147 from uDCD and 350 from DBD (171 from SCD and 179 from ECD). Positive PF cultures were significantly more frequent in the uDCD group, with 32.0% (*n* = 47) testing positive, compared to 13.7% (*n* = 48) in the DBD group (*p* < 0.001; Table 1).

**TABLE 1:** Bacteriological analysis of preservation fluid in uDCD and DBD transplants.

Microbiological status	uDCD n = 147	DBD *n* = 345	*p*-value
Culture-positive PF, *n (%)*	47 (32.0)	48 (13.7)	<0.001
*Staphylococcus*, *n (%)*			
*S. epidermidis*	21 (14.3)	18 (5.5)	
*S. lugdunensis*	10 (6.8)	6 (1.7)	
*S. capitis*	3 (2.0)	2 (0.6)	
*S. warney*	1 (0.7)	2 (0.6)	
*S. caprae*	1 (0.7)	2 (0.6)	
*S. aureus*	-	1 (0.3)	
*S. hominis*	-	1 (0.3)	
*S. haemolyticus*	-	1 (0.3)	
*Streptococcus*, *n (%)*			
*S. constellatus*	1 (0.7)	-	
*S. viridans*	1 (0.7)	-	
*S. mitis*	1 (0.7)	-	
* Escherichia coli, n (%)*	4 (2.7)	3 (0.9)	
*Enterococcus, n (%)*			
*E. faecalis*	1 (0.7)	3 (0.9)	
*E. raffinosus*	1 (0.7)	-	
*Bacillus, n (%)*			
*B. licheniformis*	-	1 (0.3)	
*B. megaterium*	-	1 (0.3)	
*Biffidus, n (%)*	1 (0.7)	-	
*Serratia marcescens, n (%)*	1 (0.7)	-	
*Corynebacterium tuberculostearicum, n (%)*	-	1 (0.3)	
*Shewanella putrefaciens, n (%)*	-	1 (0.3)	
*Klebsiella aerogenes, n (%)*	-	1 (0.3)	
*Citrobacter braakii, n (%)*	-	1 (0.3)	
*Klebsiella pneumoniae* + *Escherichia coli + Enterococcus faecium, n (%)*	-	1 (0.3)	
*Klebsiella pneumoniae* + *Escherichia coli, n (%)*	-	1 (0.3)	

DBD, donation after brain death; PF, preservation fluid; uDCD, uncontrolled donation after circulatory death.

The most commonly isolated bacteria in uDCD were *Staphylococcus* species, found in 24.5% (n = 36) of cases, followed by *E. coli* in 2.7% (n = 4). In DBD, *Staphylococcus species* was isolated in 9.6% (n = 33) of cases, with *E. coli* and *E. faecalis* both present in 0.9% (n = 3) each (Table 1).

Characteristics of patients with positive PF cultures are detailed in Table 2.

**TABLE 2:** Characteristics of patients with positive preservation fluid cultures.

Patients characteristics	uDCD with positive PF culture (*n* = 47)	DBD with positive PF culture (*n* = 48)
Donor age, years, median (P25-P75)	51.0 (41.0–57.0)	57.0 (43.3–67.0)
Donor sex, n (%) Male Female	36 (76.6) 11 (23.4)	42 (87.5) 6 (12.5)
Cause of donor death Traumatic brain injury Vascular cerebral event Cerebral hypoxia Cardiocirculatory Others	- - - 47 (100.0) -	15 (31.3) 11 (22.9) 7 (14.6) - 15 (31.3)
Date of transplant 2016-2020 2020-2024	32 (68.1) 15 (31.9)	33 (68.8) 15 (31.3)
Antibiotic prophylaxis Cephazolin Ciprofloxacin	44 (93.6) 3 (6.4)	44 (91.6) 4 (8.4)
ICU length of stay, days, median (P25-P75)	-	2 (1–3)
Time on ECMO, minutes, median (P25-P75)	180.0 (15.0–180.0)	-
CIT, hours, median (P25-P75)	13.0 (11.0–16.0)	14.5 (12.0-17.8)
Surgical extraction time, minutes, median (P25-P75)	35.0 (20–47.5)	35.0 (30.0-60.0)
Multiorgan procurement, n (%)	-	24 (50.0)
Recipient age, years, median (P25-P75)	55.0 (48.0–61.0)	59.0 (50.0-64.8)
Recipient sex, n (%) Male Female	31 (66.0) 16 (34.0)	29 (60.4) 19 (39.6)
Causes of recipient CKD, n (%) Diabetic nephropathy Hypertensive glomerulosclerosis Glomerulonephritis Urological cause Polycystic kidney disease Others Unknown	4 (8.5) 2 (4.3) 10 (21.3) 2 (4.3) 13 (27.7) 4 (8.2) 12 (25.5)	8 (16.7) 1 (2.1) 13 (27.1) - 10 (20.8) 2 (4.2) 14 (29.2)
Type of dialysis, n (%) Hemodialysis Peritoneal dialysis	39 (83.0) 8 (17.0)	41 (85.4) 6 (12.5)
Graft function, n (%) Delayed Immediate Non-function	34 (66.7) 10 (25.6) 3 (6.4)	17 (33.3) 29 (74.4) 2 (4.2)

CIT, cold ischemia time; CKD, chronic kidney disease; ECMO, extracorporeal membrane oxygenation; ICU, intensive care unit; PF, preservation fluid.

Despite the higher rate of positive PF cultures in uDCD, the incidence of bacteremia within the first week post-transplant was similar between groups, occurring in 8.5% (n = 4) of uDCD patients and 6.3% (n = 3) of DBD patients (*p* = 0.673). None of the bacteremia cases were culture-concordant with the bacteria found in the PF and none of the patients developed symptoms.

Based on PF cultures, antibiotics were administered to 10.6% (n = 5) in uDCD group compared to 22.9% (n = 11) in the DBD group (*p* = 0.110; Table 3).

**TABLE 3:** Post-transplant bacteremia and antibiotic usage associated with positive preservation fluid cultures.

Post-operative status	uDCD with positive PF culture *n* = 47	DBD with positive PF culture *n* = 48	*p*-value
Symptomatic patients, *n (%)*	0	0	
Bacteremia in first week, *n (%)*	4 (8.5)	3 (6.3)	0.673
Concordant culture	0	0	
Discordant culture	4 (8.9)	3 (6.3)	
Treatment, *n (%)*	5 (10.6)	11 (22.9)	0.110
Amoxicillin/clavulanic acid	5 (10.6) 4 - *E. coli* 1 – *E. faecalis*	7 (14.6) 2 – *E. faecalis* *2 - E.coli* 1 – E.coli + E.faecalis 1 – *K. pneumoniae + E. coli + E. faecium* *1 – S. aureus*	
Ciprofloxacin	0	1 (2.1) 1 - *K aerogenes*	
Piperacillin/tazobactam	0	2 (4.2) 1 – *K. pneumoniae + E. coli* 1 – *E. faecalis*	
Wound site infection	6 (12.8)	6 (12.5)	0.969

DBD, donation after brain death; PF, preservation fluid; uDCD, uncontrolled donation after circulatory death.

In all treated uDCD cases, amoxicillin/clavulanic acid was used, and it was the most commonly prescribed agent in the DBD group (14.6% (n = 7); Table 3).

## Discussion

PF contamination can occur at various stages of the transplantation process, particularly during procurement and packaging. Potential sources include airborne transmission within the surgical environment, surgical instruments, or inadequate skin antisepsis. Donor gut ischemia can also result in the translocation of bowel flora into the bloodstream, posing a risk to other organs [2, 9]. Additionally, the biochemical characteristics of organ preservation PF can support the growth of microorganisms [10]. In the context of multiorgan procurement, kidneys are often the last to be retrieved, increasing their exposure to these contamination risks.

Several factors can distinguish uDCD from DCD, potentially affecting PF results. uDCD involves a femoral cannulation and ECMO, an invasive intervention, which can introduce microorganisms into the bloodstream. Additionally, uDCD can occur outside of hospital settings in non-sterile environments, exposing the body to external contaminants and increasing the risk of bacterial contamination. In contrast, DBD donors are typically managed in more controlled medical environments. Moreover, warm ischemia can lead to tissue damage, rendering kidneys more susceptible to bacterial contamination. This ischemic injury may increase the permeability of the intestinal mucosa, allowing bacteria to translocate from the gut into the bloodstream and potentially contaminate the kidneys and PF. Furthermore, reperfusion injury can exacerbate tissue damage and may release bacteria from previously ischemic areas into the PF. The sudden restoration of blood flow can also disseminate bacteria that translocated during the ischemic period, increasing the bacterial load in the PF. At our center, ECD grafts are biopsied, a process that may inadvertently serve as an entry point for bacterial contamination. Additionally, the waiting period for results prolongs the time the graft remains in preservation fluid, theoretically increasing the risk of contamination.

In our study, positive PF cultures were found in 32.0% of uDCD cases, which was significantly higher than the 13.7% observed in DBD cases. Most positive PF cultures contained staphylococci, suggesting possible contamination from the donor’s skin flora.

No proven systemic infection related to PF was identified. Yu et al. reported that 2.9% of recipients developed infections that were defined as PF donor-derived infections [11]. Bacteremia was observed in 8.5% of uDCD patients and 6.3% of DBD patients with positive PF cultures, but none of the cases matched the bacteria found in the PF cultures. This finding is consistent with a previous study where no systemic infections were reported among 362 renal transplant patients [12]. According to a recent meta-analysis, despite the frequent contamination of PF in donated organs, the rate of PF-related infections remains low [10]. The lack of concordance between PF and blood cultures may indicate either that PF has a minimal role in developing systemic infections or that antibiotic treatment targeting the bacteria found in PF effectively prevents systemic dissemination. The incidence of wound site infections was comparable between the two groups.

Currently, routine culture of PF is not standard practice in most transplant centers [6, 13]. However, in France, since the 2008 guidelines from the Agency of Biomedicine, PF samples from kidney transplants are systematically collected for microbiological analysis, though no clear recommendations exist on how to manage positive bacteriological cultures from PF [14].

Despite a higher incidence of positive PF in uDCD (32.0% vs. 13.7% in DBD), antibiotics were administered to 10.6% of positive uDCD cases compared to 21.3% in the DBD group—a difference that was not statistically significant. This is likely because the predominant organisms in the uDCD group, such as *staphylococcus* and *streptococcus* species, were considered non-pathogenic and no antibiotic was given. Although some authors consider *Staphylococcus lugdunensis* to be pathogenic, no treatment was administered to the 10 patients in the uDCD group, and no related infections were observed.

One study reported that positive PF cultures resulted in antibiotic prescriptions in up to 35% of cases [15]. There is some evidence suggesting that prophylactic antibiotic use guided by PF cultures in asymptomatic patients does not reduce the rate of PF-related infections and can be part of an excessive antibiotics use [16, 17]. In other hand, some reports suggest that treating positive PF cultures can improve outcomes, including reducing infection, graft loss, and acute rejection, but only in cases involving high-risk bacteria such as Gram-negative bacilli and *Staphylococcus aureus* [10]. Despite conflicting findings, the literature identifies specific risk factors associated with positive PF cultures and the subsequent need for treatment, such as donor age, prolonged ICU stays, elevated preoperative creatinine levels, the presence of ESKAPE bacteria (*E. faecium*, *Staphylococcus aureus*, *K. pneumoniae*, *A. baumannii*, *P. aeruginosa*, and *Enterobacter* species), elevated procalcitonin levels, hemofiltration, use of sirolimus, multiorgan procurement, and en-bloc transplant procedures [10, 18–20]. *Candida* species in PF are recognized as potential causes of renal graft mycotic aneurysms [21]. While *Candida* species are known to grow on standard aerobic bacterial culture media, their identification may still be limited without the use of fungal-specific media and incubation conditions. In our study, no *Candida* species were identified, which may reflect these methodological constraints rather than their true absence. In fact, there were no reported cases of either mycotic aneurysms or post-operative candidemia.

Factors such as intestinal perforation during procurement, multiorgan procurement, and *en bloc* procurement are recognized as risk factors for PF-positive cultures [20]. In our sample, no instances of intestinal perforation were recorded during procurement, kidney *en bloc* retrieval is not performed, and only kidneys are procured in the uDCD setting. Consequently, it was impossible to assess these factors within our sample. While the use of a perfusion machine after organ procurement was suggested to reduce the risk of positive PF cultures [20], it is not used at our center, which precludes any analysis of its impact. Despite ongoing debate about the significance of bacteriological positivity in PF cultures, recent studies have explored decontamination methods using ultraviolet-C, ultrasound, and Ps80 detergent to reduce the microbial load [22]. The use of cephalosporins, such as cefazolin, for donor surgical prophylaxis can reduce the risk of organ contamination and subsequent infection in recipients. These antibiotics are renally excreted and may persist in renal tissue after procurement, providing continued antimicrobial protection [2]. The rate of positive PF cultures was higher during the first 4 years of the program, possibly due to the learning curve or procedural evolution.

Given the low treatment rates and minimal clinical impact, our findings support the literature questioning the necessity of routinely performing bacteriological analysis of PF. Minimizing antibiotic overuse is essential, particularly in kidney transplant recipients who are inherently vulnerable to multi-resistant bacterial infections. Currently, antibiotic prescription is based primarily on clinical judgment, leading to considerable variation between centers, and there are no universal guidelines for collecting PF cultures, determining the timing of collection before transplantation, selecting specific treatments, or monitoring recipients in the event of a positive PF culture.

The impact of the length of stay in intensive care as a risk factor for PF contamination could not be reliably assessed between groups, as uDCD donors do not stay in the ICU; instead, they are placed on ECMO and taken directly to the operating room.

Anaerobic cultures were not conducted in this study, consistent with many reports in the literature that do not routinely screen for anaerobes. The limited available data indicates a low incidence of infections caused by these agents [17, 20]. Transplants from controlled DCD were not included, as this type of transplantation is not regulated in the country. Patients who underwent early surgical reintervention were also excluded to avoid potential data misinterpretation due to additional contamination of the surgical site and blood cultures. The study’s single-center design may limit generalizability. While the overall sample is robust, some subgroup analyses were conducted with relatively small sample sizes, which may reduce statistical power. The available literature primarily addresses other kidney transplantation donation settings, making direct data comparisons challenging, as this appears to be the first prospective publication focused on the incidence of positive microbiological cultures in PF from uDCD kidney transplants compared to those from DBD and its clinical impact. Future research should examine the differences in PF cultures between kidneys from the same donor and compare outcomes from multi-organ uDCD procurement with those from kidney-only procurement. Collecting culture samples immediately after harvesting could be important for comparison with samples taken at the time of transplant. Excluding patients who required surgical reintervention within the first 8 days post-transplant may have inadvertently omitted cases of donor-derived anastomotic infections, which are often associated with high-risk pathogens in PF cultures. This exclusion could introduce bias and limit the generalizability of the findings. The authors justified this decision by noting that reintervention may alter microbiological results, particularly blood cultures. Notably, among the cases requiring graft removal during early reintervention, no association was found between pathogenic organisms and histopathological analyses. The study’s ability to assess the impact of high-risk pathogens is limited, as most peritoneal fluid cultures did not yield such organisms. However, this also suggests that the prevalence of these high-risk microorganisms is low.

In conclusion, PF contamination is common in kidney transplantation, particularly in the uDCD setting. Although positive PF cultures were more frequent in uDCD cases, most of the bacteria identified were non-pathogenic and antibiotic prescription rates were similar compared to the DBD group.

## Data Availability

The raw data supporting the conclusions of this article will be made available by the authors, without undue reservation.
