# Corrigendum: Preservation Fluid Bacteriology in Kidney Transplantation: Comparing Uncontrolled Donation After Circulatory Death With Donation After Brain Death

**DOI:** 10.3389/ti.2025.15389

**Published:** 2025-09-23

**Authors:** Alberto Costa Silva, Teresa Pina-Vaz, Ana Pinho, Inês Ferreira, Ana Cerqueira, Manuela Bustorff, Susana Sampaio, Roberto Roncon-Albuquerque, Margarida Rios, Manuel Pestana, Carlos Martins-Silva, Tiago Antunes-Lopes, João Alturas Silva

**Affiliations:** ^1^ Urology Department, University Hospital Center of São João, Porto, Portugal; ^2^ Faculty of Medicine, University of Porto, Porto, Portugal; ^3^ RISE- Health Research Network, Faculty of Medicine, University of Porto, Porto, Portugal; ^4^ Nephrology Department, University Hospital Center of São João, Porto, Portugal; ^5^ Intensive Care Unit, University Hospital Center of São João, Porto, Portugal; ^6^ Transplant Coordination Office, University Hospital Center of São João, Porto, Portugal

**Keywords:** kidney transplantation, organ procurement, preservation fluid, donation after circulatory death, infection

In the published article, there was a missing **Funding** statement. The correct **Funding** statement appears below.

